# Impaired belief updating and devaluation in adult women with bulimia nervosa

**DOI:** 10.1038/s41398-022-02257-6

**Published:** 2023-01-06

**Authors:** Laura A. Berner, Vincenzo G. Fiore, Joanna Y. Chen, Angeline Krueger, Walter H. Kaye, Thalia Viranda, Sanne de Wit

**Affiliations:** 1 Department of Psychiatry, Icahn School of Medicine at Mount Sinai, New York, NY USA; 2 Department of Psychology, Drexel University, Philadelphia, PA USA; 3 Department of Psychiatry, University of California San Diego, San Diego, CA USA; 4 Department of Clinical Psychology, University of Amsterdam, Amsterdam, Netherlands

**Keywords:** Psychiatric disorders, Human behaviour

## Abstract

Recent models of bulimia nervosa (BN) propose that binge-purge episodes ultimately become automatic in response to cues and insensitive to negative outcomes. Here, we examined whether women with BN show alterations in instrumental learning and devaluation sensitivity using traditional and computational modeling analyses of behavioral data. Adult women with BN (*n* = 30) and group-matched healthy controls (*n* = 31) completed a task in which they first learned stimulus-response-outcome associations. Then, participants were required to repeatedly adjust their responses in a “baseline test”, when different sets of stimuli were explicitly devalued, and in a “slips-of-action test”, when outcomes instead of stimuli were devalued. The BN group showed intact behavioral sensitivity to outcome devaluation during the slips-of-action test, but showed difficulty overriding previously learned stimulus-response associations on the baseline test. Results from a Bayesian learner model indicated that this impaired performance could be accounted for by a slower pace of belief updating when a new set of previously learned responses had to be inhibited (*p* = 0.036). Worse performance and a slower belief update in the baseline test were each associated with more frequent binge eating (*p* = 0.012) and purging (*p* = 0.002). Our findings suggest that BN diagnosis and severity are associated with deficits in flexibly updating beliefs to withhold previously learned responses to cues. Additional research is needed to determine whether this impaired ability to adjust behavior is responsible for maintaining automatic and persistent binge eating and purging in response to internal and environmental cues.

## Introduction

Bulimia nervosa (BN) is a disabling disorder characterized by recurrent episodes of eating that feel out of control and compensatory behaviors, including self-induced vomiting, exercise, or laxative misuse [1]. Over 60% of patients who receive first-line treatments for BN remain symptomatic [2], and very little is known about the neurocognitive mechanisms that may contribute to these entrenched symptoms.

Multiple theories have posited a role for conditioning to antecedent cues in BN. These theories propose that particular internal and external cues may start to function as conditioned stimuli that prompt the conditioned response of binge-eating and purging urges and behaviors [3]. Consistent with this notion, individuals with BN and those with binge-eating disorder (BED; who binge eat but have no compensatory behaviors) show greater increases in food craving after exposure to visual food cues than do healthy adults without eating disorders [4]. In addition, internal cues, like negative affective states, have been shown to reliably precede, and potentially prompt bulimic behaviors [5]. The repeated pairing of these stimuli (S) and behavioral responses (R) may lead to the formation of S-R associations, such that these behaviors may become increasingly automatic, habitual, and ultimately compulsive [6]. However, the precise cognitive mechanisms that may go awry to promote persistently strong links between cues and behavior in BN are poorly understood.

Some data suggest that an altered ability to engage goal-directed control to stop engaging in behaviors that are no longer valuable may play a role. This ability is impaired in other impulse-control disorders [7, 8], and findings from rodent models indicate that over time, repeated episodes of binge-like food consumption results in a failure to reduce responding for devalued food rewards [9]. In line with these preclinical results, individuals with BED show a bias towards “model-free” (as opposed to “model-based”) behavior on a two-step decision-making task, which has been argued to reflect a tendency to rely on automatic or habitual responses instead of goal-directed responses that are sensitive to outcomes [10]. As such, an impaired ability to flexibly adjust behavior in a goal-directed manner when outcomes are no longer valuable may contribute to binge eating. Such an impairment could contribute to the transition from repeated binge eating and purging to a treatment-resistant disorder, where binge-eating and purging behaviors persist despite negative consequences like guilt, shame, affective instability, and health risks [11, 12].

However, behavioral adjustment is required not only after receiving new information about the value of the behavior’s outcome, but also after receiving new information about the value of the cue that precedes the behavior. Some theories posit that overvaluation of cues or stimuli that tend to precede initially rewarding behaviors such as substance use, gambling, and overeating, could promote persistent engagement in these behaviors [13]. As such, alterations in the complex process of updating and learning about links between stimuli and behaviors (i.e., stimulus-response associations) may also contribute to entrenched bulimic symptoms. Some data suggest that individuals with BN show altered value learning and stimulus-response learning [14–16], yet very little research to date has focused on how individuals with BN adjust, or fail to adjust, their responses when the value of associated stimuli changes. An impaired ability to adjust behavior when the values of discriminative stimuli change could result in automatic bulimic behaviors in response to particular cues (e.g., binge foods, strong negative emotion) that persist despite efforts to respond in new ways to those cues (e.g., using an adaptive emotion regulation skill instead).

Here, we investigated instrumental learning and the ability to adapt to temporary devaluation of stimuli or outcomes in 30 women with BN and 31 group-matched healthy controls. We used a multi-stage task that includes an instrumental learning phase and a test of response-outcome learning. Finally, participants were instructed to withhold responses to stimuli when their linked outcomes are no longer valuable (i.e., outcome devaluation) in the “slips-of-action test” or to withhold responses to specific stimuli (i.e., stimulus devaluation) in the “baseline test.” Both of these final tests require intact working memory and inhibitory control, but unlike the slips-of-action test, the baseline test does not require any recall of information about outcomes in order to select the correct response (i.e., outcome retrieval). This task has been used previously to study individuals with anorexia nervosa (AN; [17]) and autism spectrum disorders [18], who showed no differences from healthy controls; individuals with unmedicated Tourette’s syndrome, who showed deficits in outcome devaluation that were associated with tic severity [19]; individuals with obsessive-compulsive disorder (OCD) [8] and individuals with obsessive-compulsive symptoms [20], who showed deficits in outcome devaluation [8] that were associated with compulsion severity [20]; and adults with a history of depression, whose number of past depressive episodes correlated with lower outcome-devaluation sensitivity [21].

We analyzed participants’ behavior using both traditional methods and computational modeling to identify the underlying cognitive processes characterizing the two groups. In traditional analyses, we predicted that, compared with healthy controls (HC), women with BN would demonstrate deficits in goal-directed control in the slips-of-action test, evidenced by a persistence of learned responses despite devaluation of their outcomes. In computational analyses, we developed and fitted a model based on Bayesian inference to estimate belief updating across all four phases of the task. For the instrumental learning phase of the task, we compared the performance of the Bayesian model with a reinforcement-learning model previously applied to a similar task in addiction [22]. We compared BN and HC groups on model parameters from the winning model for the first phase of the task and the Bayesian model for the final two phases. Exploratory analyses investigated associations of task behavior with bulimic symptoms.

## Materials and methods

### Participants

Participants were right-handed [23] females aged 18 to 35 and weighing between 85 and 120% of the expected weight for their height based on the Metropolitan Life Insurance tables [24]. Women with BN met *DSM-5* criteria (at least one objective bulimic episode and compensatory behavior per week for the past three months)[1], purged via self-induced vomiting (though other methods could additionally be endorsed; see Table 1), and if they were taking psychoactive medications, were on a stable dose of all psychoactive medications for at least 4 weeks before study. Healthy controls were excluded if they (1) met criteria for the diagnosis of any Axis I psychiatric disorder in their lifetime; (2) had any history of eating disorder behavior, or (3) used psychoactive or other medication known to affect mood or concentration in the last 3 months. Women with BN were excluded if they had any comorbid Axis I disorder apart from major depression, generalized anxiety disorder, social anxiety disorder, or panic disorder (see Supplement for full inclusion and exclusion criteria). Participants were recruited from the UC San Diego Eating Disorders Center for Treatment and Research and the San Diego community (see Table 1 for treatment status information).

**Table 1:** Sample characteristics and state comparisons.

	Healthy controls *N* = 31	Bulimia nervosa *N* = 30		
	*M* (SD) or *n (%)*	*M* (SD) or *n (%)*	*t, W, or χ*^*2*^	*p* value
Demographics				
Age (years)	22.6 (2.9)	22.6 (3.6)	0.023	0.982
Body mass index (kg/m^2^)	21.9 (1.8)	21.9 (2.2)	0.120	0.905
Full scale IQ score	108.4 (10.0)	107.1 (11.6)	0.453	0.653
Years of education	15.6 (1.7)	15.0 (1.9)	1.243	0.219
Self-reported race	–	–	0.447	0.504
Hispanic	6 (19.4)	3 (10)	–	–
Self-reported ethnicity	–	–	3.972	0.265
White	15 (48.4)	18 (60.0)	–	–
Black/African American	0	0	–	–
Asian	10 (32.3)	11 (36.7)	–	–
Pacific Islander	0	0	–	–
American Indian/Alaska Native	1 (3.23)	0 (0)	–	–
Other race	5 (16.1)	1 (3.3)	–	–
Eating disorder symptoms				
Eating Loss of Control Scale severity score	0.6 (0.5)	6.5 (1.8)	6.5	<0.001
Objective bulimic episodes (past 3 months)	–	49.5 (37.6)	–	–
Self-induced vomiting (past 3 months)	–	59.8 (48.4)	–	–
Diuretic misuse episodes (past 3 months)	–	4.3 (17.2)	–	–
Laxative misuse episodes (past 3 months)	–	6.5 (25.9)	–	–
Driven and compulsive exercise days (past 3 months)	–	32.6 (28.8)	–	–
Other compensatory behavior days (e.g., chewing and spitting; past 3 months)	–	2.4 (7.3)	–	–
Comorbidities and treatment				
Major depressive disorder	–	8 (26.7)	–	–
Anxiety disorder	–	8 (26.7)	–	–
Generalized anxiety disorder	–	4 (13.3)	–	–
Social anxiety disorder	–	7 (23.3)	–	–
Past anorexia nervosa	–	14 (46.7)	–	–
Hormonal birth control	14 (46.7)	15 (50)	0.01	0.903
Behavioral treatment^a^	–	9 (30)	–	–
Psychotropic medication^b^	–	5 (16.7)	–	–
State before the instrumental learning and devaluation task				
Time since last meal (minutes)	361.0 (315.8)	343.7 (268.4)	218.5	0.322
Time since last menstrual period (days)^c^	9.48 (13.4)	16.7 (16.0)	228.5	0.005
Hunger^d^	20.4 (11.4)	16.9 (10.9)	539.0	0.185
Fullness^d^	20.6 (12.4)	21.2 (12.1)	439.0	0.875
Desire to binge eat^d^	–	23.8 (21.9)	–	–
Desire to purge^d^	–	17.9 (20.1)	–	–

^a^In the bulimia nervosa group, nine women were receiving behavioral treatment (*n* = 6 outpatient psychotherapy, *n* = 3 partial hospitalization).

^b^In the bulimia nervosa group, five women were taking psychotropic medication at a stable dose for at least 4 weeks (*n* = 1 on escitalopram, *n* = 1 on fluoxetine, *n* = 1 on fluoxetine and gabapentin, *n* = 1 on venlafaxine; *n* = 1 on alprazolam pro re nata (PRN), but abstained from taking this PRN medication in the week prior to study.

^c^Four participants with bulimia nervosa reported that they had not menstruated in over 3 months. Reported results reflect means and group comparisons of individuals who reported menstruating in the past 3 months (*n* = 26 women with bulimia nervosa).

^d^These ratings were collected using the generalized Labeled Magnitude Scale [55].

Individuals were screened and characterized using the Mini-International Neuropsychiatric Interview (M.I.N.I.; [25]) and the Structured Clinical Interview for *DSM-5* (SCID-5; [26]; see Supplement for further detail), and diagnostic items of the Eating Disorder Examination (EDE; [27]) established BN diagnosis and symptom frequencies. The two-subtest Wechsler Abbreviated Scale of Intelligence (WASI-II; [28]) estimated general intellectual functioning (FSIQ). Participant characteristics are presented in Table 1. All participants provided written informed consent, and the University of California San Diego’s Human Research Protections Program approved the protocol. The study was registered on ClinicalTrials.gov (NCT02997475).

### Instrumental learning and devaluation paradigm

**Fig. 1:**
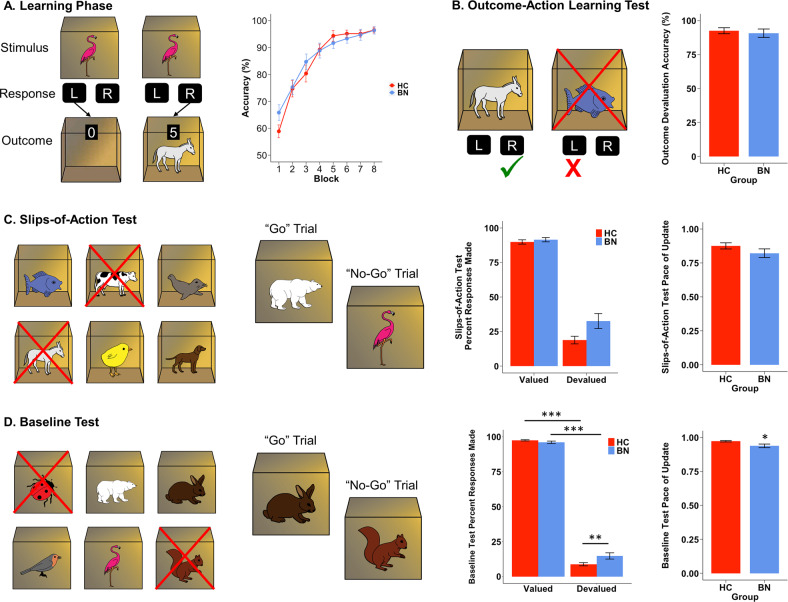
The instrumental learning and devaluation task. **A** In an instrumental learning phase, participants learn through feedback to associate stimuli with responses and outcomes. A total of six different stimuli and six different outcomes were used. Stimulus–response associations (e.g., the flamingo-labeled box could be opened with the right button) and stimulus–outcome associations (e.g., the flamingo-labeled box had a donkey inside as the outcome) were counterbalanced across participants. This first phase included six blocks of 12 trials each. Within blocks, each stimulus appeared twice in randomized order. All participants’ performance improved over time, indicating learning, but groups did not differ in this improvement. **B** The subsequent outcome-action learning test assesses associations of responses and outcomes that were learned in the first phase of the task. On each trial, two open boxes with animals inside were presented (one that was previously earned by a left key press and one by a right key press), but one had a cross over it to indicate that it was no longer worth points. On each trial, participants were instructed to press the key that led to the still-valuable animal on the inside of the box (the animal that was not crossed out). All outcomes were crossed out twice over the course of this test. Women with bulimia nervosa did not differ from controls on this test of outcome-action knowledge. **C** The “slips-of-action” test measures the relative balance of goal-directed vs. habit-based action by the ability to respond only to still-valuable outcomes (percent responses to still-valuable stimuli versus percent responses for now devalued stimuli). Participants are explicitly and repeatedly (after every 12 trials; 9 times total) told which outcomes are no longer valuable, so the behavior that previously lead to that outcome should no longer be enacted. Each of the six outcomes were devalued three times across the blocks. The test was performed in “nominal extinction,” meaning that neither outcomes nor points earned or lost were shown. Participants were instructed, however, that their final score would be shown at the end of the task. There was no group x valuation interaction detected on this test, and groups did not differ on the computational parameter (λ) that indexes the pace of belief update on this test. **D** The “baseline” test is identical to the slips-of-action test, except that stimuli, instead of outcomes, are devalued. Participants are explicitly and repeatedly told which stimuli no longer lead to rewards, so those stimuli should no longer be responded to. A group x valuation interaction indicated that women with bulimia nervosa responded significantly more to devalued trials on this subtest, and their lower λ values indicated that their pace of belief update was slower during the baseline test. HC healthy controls, BN women with bulimia nervosa, post-hoc pairwise comparisons: *p < 0.05, **p < 0.005, ***p < 0.001.

Participants completed a four-phase instrumental learning and devaluation task that assesses the ability to engage goal-directed control to override previously learned stimulus-response mappings after outcome and stimulus devaluation [17, 29]. In the first phase of the task, instrumental learning (Fig. 1A), participants learned by trial and error to open boxes with different animal images on them (discriminative stimuli), using left or right button presses (responses). Correct responses resulted in rewards, consisting of different animal images inside each box (outcomes) and points. No reward was given if the incorrect button was pressed. Faster correct responses within the 2-s response window led to more points being awarded [30]. Of note, this was the only phase of the task that included any feedback. Participants were told to make note of which animals appeared on the inside of the box, as they would be tested on this information in later phases of the task. The next phase of the task (Fig. 1B) assessed for response-outcome knowledge learned in the first phase of the task, as well as the ability to use action-outcome knowledge to direct responses toward still-valued outcomes and away from devalued outcomes. On each trial, two open boxes with animals inside were presented with one crossed out. Participants were instructed to press the key that they had learned led to the animal that was not crossed out.

Subsequently, the slips-of-action test (Fig. 1C) and baseline test (Fig. 1D) were administered in counterbalanced order. Both tests included 9 blocks of 12 trials. In the slips-of-action test, a 10-s screen preceding each block of trials indicated which two of the six outcomes were devalued by marking them with a superimposed red cross. Participants were instructed to then respond as quickly as possible to stimuli associated with a still-valued outcome (thereby gaining points) and to avoid responding to those associated with devalued outcomes (which would cost them points).

The baseline test was identical to the slips-of-action test except that stimuli (outside-of-the-box animals) were devalued, instead of outcomes (inside-of-the-box animals). Similar to the slips-of-action test, every 12 trials, new instructions indicated which two new stimuli would no longer lead to rewards, so those stimuli should no longer be responded to. This test was designed to control for general task characteristics of the slips-of-action test, such as having to remember which cues are devalued and having to refrain from responding. However, unlike the slips-of-action test, this test does not require consideration of the specific anticipated outcome of a response and does not include outcome-retrieval demands. In addition, unlike a traditional response inhibition task, the baseline test requires participants to repeatedly (nine times over the course of the test) update which of six cues require a no-go response and to suppress previously learned stimulus-response associations [30].

Finally, explicit knowledge of the learned associations between stimuli and responses (stimulus-response contingencies), responses and outcomes (response-outcome contingencies), and stimuli and outcomes (stimulus-outcome contingencies) was assessed after the completion of the task via paper-and-pencil questionnaires administered in counterbalanced order. A score of 6 indicates perfect knowledge for each contingency type.

### Go/no-go task

Because both the slips-of-action and the baseline tests require the ability to successfully withhold responses (inhibitory control), which has been shown to be impaired in individuals with BN [31], exploratory sensitivity analyses examined performance on a go/no-go paradigm [32–34] in which responses to no-go stimuli (commission errors) serve as a key measure of deficits in action restraint [35]. On this task, no stimulus-response updating is required—participants are explicitly told that they should always withhold responses to upward-pointing arrows and respond to arrows pointing left or right (see Supplement).

### Traditional analyses of behavior

Hierarchical linear mixed effects models (LMEs) conducted in R [36] with subject as a random effect compared groups on accuracy across the 8 blocks of the instrumental learning phase and assessed the significance of group × valuation interactions for responses on the baseline and slips-of-action tests. Although performance on the outcome-action learning test was not of a priori interest, exploratory Wilcoxon Rank Sum Tests compared groups on accuracy in this phase of the task.

### Computational modeling of behavior

We used two computational models. The first model is based on reinforcement learning (RL) algorithms, and it estimates subject-specific values assigned to stimulus-response contingencies. The second model, based on Bayesian inference, estimates subject-specific beliefs about the probability that a response to a particular stimulus is optimal. Therefore, while the RL model focuses on learning stimulus-response values based on experienced outcomes, the Bayesian model focuses on updating beliefs. Within this Bayesian framework, the process of belief updating is based on the participant’s assumptions about how likely it is that a particular behavior in response to a particular stimulus will yield a desired outcome. If this probability is high (i.e., close to certainty), beliefs are immediately updated, whereas if this probability is low, beliefs are slowly updated.

#### Reinforcement learning model

Consistent with a prior study using a similar paradigm [22], we used a standard RL model [37] to simulate participants’ instrumental learning behavior during the first phase of the task. This was the only phase that provided an explicit valued-based outcome (*O*) after each choice selection, allowing the model to assign and update values to stimulus-response choice selections. To update the values associated with the available motor responses (left/right button press) for each of the six input stimuli (*j*), the model relied on prediction-error (PE) estimations. Trial-by-trial PEs were then used to update incrementally the subjective values for the next trial (*V*_*t*+1_), given the current input animal cue and chosen action, by adding the expected value from the current trial (*V*_*t*_) to the computed PE, multiplied by a coefficient (*α*), which determined the learning rate or sensitivity to the PE, as follows:

1 $$V_{j,t + 1} = V_{j,t} + \alpha \left( {O_t - V_{j,t}} \right)$$

Because faster responses were awarded more points, we tested two potential computations of the PE (*O*_*t*_-*V*_*j,t*_): one with a variable, reaction time-dependent outcome (between 0 and 5), and one with a fixed trial-by-trial outcome (always 5), as in [22] (see Supplement).

To transform values into choice selection probabilities [*p*(left_t_) and *p*(right_t_)], we used a softmax function adapted to account for subject-specific differences in perseverative behavior (captured by *τ*) and exploratory behavior (captured by *β*), which could vary dynamically during the task ([22]; see Supplement).

#### Bayesian model

Despite the strengths of the RL model in capturing information about value updating, this algorithm cannot model behavior past the first phase of the task. This is because subsequent phases of the task rely on explicit instructions concerning the stimuli or outcomes that have been devalued, and offer no feedback that could be used to inform the correctness of a response (i.e., the value associated with an action selection).

To overcome these limitations, we used a Bayesian model of belief updating that relied on categorical evidence (i.e., input/output cues and devaluation), rather than valued outcomes, to generate and update beliefs about the associations among input stimuli, choice selections, and output stimuli. Since this model does not require explicit feedback, it could be applied to all four phases of the task.

The model estimated the subject-specific, trial-by-trial subjective beliefs about input-action-outcome associations (i.e., Bayesian inference) and outcome-action-input associations (i.e., Bayesian inversion). We estimated, on each trial (t) and for each input cue (*in*_j_), each subject’s estimated probability that performing an action or not (*a*_*go*_ *or a*_*no-go*_) would yield a rewarded output cue (*out*_*j*_), and, if an action was performed, the probability that pressing left versus right (*a*_*left*_ *or a*_*right*_) would yield a rewarded output cue (*out*_*j*_). Prior beliefs were incrementally updated into posteriors according to Bayes rule,

2 $$P_{t + 1,j}(a_i) \propto P\left( {out_j|a_i} \right)P_{t,j}\left( {a_i} \right)$$

where subject-specific assumptions about the likelihood λ = *P*(*out*_*j*_|*a*_*i*_) determined a subject-specific pace of belief update (see Supplement for further details).

In the baseline and slips-of-action tests, we assumed a decay of the beliefs from the end of the learning acquired in the first task phase, identical for all subjects (see Supplement). These priors were then updated based on the instruction screen for each block.

After the best-fitting model parameters were identified per subject, Wilcoxon Rank Sum Tests were used to compare groups on parameters from the RL and Bayesian models. In the final two phases of the task, analyses concentrated on the λ parameter regulating the updating of beliefs about whether a response should be made.

### Exploratory analyses

#### Associations with symptoms

As in prior research [17, 18, 20, 21, 38], we calculated a devaluation sensitivity index (DSI) for both the slips-of-action and baseline tests by subtracting the percentage of responses to devalued cues from the percentage of responses to still valuable cues. Exploratory negative binomial regressions examined associations of binge eating and self-induced vomiting frequencies in the last 3 months with these DSIs and with parameters from computational models.

#### Sensitivity analyses

Sensitivity analyses examined potentially confounding effects of comorbid anxiety and depression, medication status, history of anorexia nervosa (AN); and days since last menstrual period. Additional sensitivity analyses compared groups on inhibitory control ability as measured by go/no-go task commission errors and related these errors to baseline test performance.

## Results

### Traditional analyses of behavior

As reflected by the main effect of block on accuracy in the first task phase, all participants learned the association between stimuli and responses over time (Fig. 1A; *B* = 4.50, SE = 0.22, *p* < 0.001). By the final block, mean accuracy was 96.5% (SD = 6.0%) in the HC group and 96.4% (SD = 7.5%) in the BN group. Adding the group effect to accuracy models did not improve fit, and adding a group x block interaction significantly worsened (*p* = 0.044) model fit.

Subsequently, groups performed at the same high level on the outcome-action learning test (*p* = 0.873 for main effect of group on accuracy; Fig. 1B). Moreover, self-report measures of explicit recall of stimulus-response, response-outcome, and stimulus-outcome associations indicated that groups remembered the information they acquired in the first phase of the task equally well (Table 2).

**Table 2:** Explicit stimulus-response-outcome knowledge.

	Healthy controls	Bulimia nervosa	*Est (W)*	*p* value
	*M* (SD)	*M* (SD)		
S-R score	5.9 (0.4)	5.7 (0.6)	498.0	0.252
R-O score	5.5 (0.9)	5.1 (1.2)	542.5	0.112
S-O score	5.1 (1.2)	4.8 (1.6)	470.5	0.733

*S-R* stimulus-response, *R-O* response-outcome, *S-O* stimulus-outcome.

On the slips-of-action test, a group × valuation interaction did not improve the fit of the model predicting the percent of responses made (*χ*^2^(1) = 3.66, *p* = 0.056; group × valuation fixed effect *p* = 0.061; Fig. 1C). Of note, visually apparent group differences on the slips-of-action test (Fig. 1C) were considerably influenced by two women with BN who responded to 100% of devalued trials, suggesting that they may not have understood the slips-of-action test. However, these participants’ performance did not indicate a misunderstanding of the baseline test instructions (they responded to only 8% and 33% of devalued baseline test trials, respectively). In light of these outliers, we (1) repeated the LME model excluding these participants and (2) conducted a robust repeated-measures analysis of variance using the WRS2 package in R and found, in both cases, that slips-of-action group × valuation interaction effects remained non-statistically significant (*p* = 0.174 and *p* = 0.418, respectively). However, on the baseline test, a group × valuation interaction significantly improved model fit (*χ*^2^(1) = 7.54, *p* = 0.006; group × valuation fixed effect: *B* = 7.37, SE = 2.69, *t* = 2.74, *p* = 0.007, *R*^*2*^_*β*_ = 0.06). Similarly, an exploratory robust repeated-measures analysis of variance for the baseline task replicated findings from the mixed-effects model, as the group × valuation interaction effect was statistically significant (*p*  =  0.039). Post-hoc pairwise comparisons indicated that both groups responded more to valued than devalued trials (*p*s < 0.001), and while groups did not differ on responses to still-valuable stimuli (*p* = 0.454), the BN group responded more frequently to devalued stimuli (*p* = 0.002; Fig. 1D).

### Computational modeling

#### Stimulus-response-outcome learning phase: Reinforcement learning model

The RL model was a better fit to the first phase of the task than the Bayesian model was for all subjects. As such, computational analyses of the first task phase focused on parameters from the RL model. The BN group did not differ from HC on any RL model parameters (*p*s > 0.120).

#### Slips-of-action and baseline tests: Bayesian model

There were no between-group differences in the go/no-go pace of update parameter from the slips-of-action test (*p* = 0.340; Fig. 1C), but women with BN showed a slower pace of update (i.e., smaller λ) in the baseline test (*W* = 610, *p* = 0.036; Fig. 1D).

As a cross-check of the λ parameters, we explored whether lower values, which would indicate increased uncertainty and a slower pace of update, were associated with slower responses. Huber robust regressions confirmed that across groups, a slower pace of update predicted a slower reaction time on valuable trials of both the baseline test (*z* = −1028.73, *p* < 0.001) and the slips-of-action test (*z* = −259.93, *p* = 0.019).

### Exploratory post-hoc analyses

We explored whether the slips-of-action test may have prompted more deliberation (as potentially indicated by slower responding) than the baseline test did. Indeed, LME models indicated that participants responded more slowly during the slips-of-action test than on the baseline test (see Supplement). In addition, to explore whether BN may be characterized by a generalized strong reliance on learned stimulus-response associations, we followed the approach of a prior study using a similar task in OCD [39]: We collapsed performance across the baseline and slips-of-action test. A group x valuation interaction indicated that women with BN were less sensitive than HC to devaluation in general (of stimuli or outcomes; see Supplement). However, to further explore whether the baseline test assesses a uniquely altered process in women with BN, we conducted within-group robust regressions relating the DSIs from the baseline and slips-of-action tests. As in past studies of healthy participants [21] these DSIs were correlated in HCs, but they were unrelated in the BN group (see Supplement).

### Exploratory associations with symptoms

#### Devaluation sensitivity index

Lower baseline-test DSI was associated with more frequent binge eating (*z* = −2.38, *p* = 0.018) and more frequent self-induced vomiting (*z* = −2.78, *p* = 0.005) in the past 3 months. In contrast, slips-of-action-test DSI was unassociated with binge eating (*p* = 0.074) or vomiting (*p* = 0.255).

#### Computational parameters for devaluation

A slower pace of update in the baseline test was associated with more frequent binge eating (*z* = −2.53, *p* = 0.012) and self-induced vomiting (*z* = −3.09, *p* = 0.002) in the past 3 months. The slips-of-action test pace of update was not associated with bulimic symptoms (*p*s > 0.070).

### Exploratory sensitivity analyses

#### Potential clinical confounds

Results of sensitivity analyses are presented in the Supplement. Task performance was unrelated to the number of days since last menstruating, and results were unchanged when accounting for history of AN, medication status, or a comorbid anxiety disorder. Analyses excluding women in the BN group with major depression (*n* = 8) indicated that there were still group x valuation effects in traditional analyses of behavior on the baseline test, but group differences in baseline-test pace of update were no longer statistically significant. Group differences in pace of update on the slips-of-action test remained non-statistically significantly different when those with depression were excluded, but group × valuation effects in traditional analyses of behavior on the slips-of-action test became statistically significant (*p* = 0.048).

#### Inhibitory control

One participant with BN was excluded from go/no-go task analyses as she responded to only 32.5% of go trials. The BN group did not show statistically significant deficits relative to controls as indexed by commission errors (*p* = 0.087). In addition, in the BN group, commission error rate was unrelated to DSI, responses to devalued stimuli, or the pace of update parameter on the baseline test (*p*s > 0.650).

## Discussion

This is the first study to apply computational modeling to data from an instrumental learning and devaluation task to test for potential deficits in behavioral adaptability in BN. This task measures the ability to change behaviors after the outcomes that follow them or the cues that preceded them have been devalued, and it has been widely used to assess behavioral flexibility in other clinical populations. Results indicated that healthy women and women with BN were equally able to use feedback to learn associations among stimuli, responses, and outcomes. Counter to our hypotheses, women with BN did not show excessive responding towards devalued outcomes. However, when directly instructed to withhold learned responses to specific stimuli, women with BN were more vulnerable to errors. Our computational modeling results suggest that this aberrant behavior may be driven by maladaptive belief updating, which, in BN, resulted in a slower pace of behavioral adjustment (i.e., lower values for the λ parameter) when participants were presented with new information about the value of stimuli. In addition, increased responding to devalued versus valued stimuli and the parameter λ were both linked to symptom severity. Demonstrating the specificity of our findings, explicit stimulus-outcome knowledge, response-outcome knowledge, and stimulus-response knowledge at the end of the task were intact in the BN group, and the BN group did not show statistically significant deficits relative to controls on a separate inhibitory control task with static stimulus-response mappings. As such, it is unlikely that more frequent responses to devalued stimuli could be accounted for by group differences in encoding, working memory, or inhibitory control abilities. Taken together, these findings suggest that BN is characterized by perseverative responses after direct instruction to override learned stimulus-response associations, this perseveration may be underpinned by a slow pace of belief update after receiving new information about stimulus value, and these alterations may contribute to binge eating and purging in BN.

Consistent with some [15, 40], but not all [41], prior research in adults with BN, both traditional and computational modeling analyses of behavior from the first two phases of the task, as well as post-task questionnaires, indicated that individuals with BN were able to learn the associations of stimuli, responses, and outcomes as well as HC. Therefore, although food-specific reward-learning may be altered in BN [16], our findings add to growing evidence suggesting that BN is not characterized by generalized reward-learning deficits. Moreover, in line with results from individuals with AN and autism using the same task and from individuals with obesity using a food-specific version of the task [17, 18, 42, 43], our results from the slips-of-action test suggest that BN is also characterized by an intact ability to exert goal-directed control when outcomes are explicitly devalued.

However, individuals with BN showed deficits when stimuli were devalued, suggesting difficulties in flexibly suppressing acquired stimulus-response associations. Our Bayesian model indicated that this difficulty may be attributed to impaired belief updating. Consistent with the notion that persistently strong links between cues (e.g., the sight of binge foods, negative affect) and responses contribute to the maintenance of bulimic symptom urges and behaviors [6], these impairments were most pronounced in individuals with the most frequent binge eating and self-induced vomiting. Our findings also align with prior results indicating cognitive inflexibility, perseverative behaviors, and set-shifting deficits in BN [44, 45]. In addition, our results supporting deficits in stimulus devaluation but not outcome devaluation are consistent with initial results in BN from a two-stage task estimating “model-free” and “model-based control,” the latter of which has been shown to correlate with the slips-of-action test DSI [38]. On this two-stage task, adults with BN did not show deficits in model-based control, but instead showed alterations in model-free, stimulus-response-based performance [46].

Since both the slips-of-action and baseline tests require inhibition of previously learned responses when presented with stimuli, a prior study using a similar task collapsed performance across tests and found an overall increase in responses to devalued trials in adolescents with OCD [39]. Our analogous exploratory analysis indicated a similar overall increase in BN. Thus, as was suggested for adolescent OCD, the presentation of stimuli may more strongly trigger previously learned responses in women with BN than in HC. However, further suggesting that BN may be associated with a unique alteration in belief updating when the value of conditioned *stimuli* change, whereas the DSI and λ parameter from the baseline test were associated with binge-eating and purging severity, DSI and the λ parameter from the slips-of-action test were unrelated to symptom severity. In addition, performance on the slips-of-action test and the baseline test were unrelated in individuals with BN.

There are several potential reasons why we detected group differences on the baseline test but not the slips-of-action test. First, there are important differences in the instructions and updating required in the tests. The baseline test relies on standard Bayesian inference processes: stimulus-response-outcome chains need to be updated depending on the new available information about the first element in the chain. Conversely, because outcomes in the slips-of-action test are devalued by explicit instruction, not by classic reversal learning exposures (i.e., by experiencing stimulus-response-devalued outcome links), this test requires updates via Bayesian inversion (i.e., outcome-response-stimulus). Thus, BN might be more strongly associated with deficits in updating beliefs that are more easily accessible and, we speculate, perhaps closer to a classic habitual or automatic behavioral strategy.

Second, and related to these distinct updates, the cognitive demand and deliberation required in the slips-of-action test may be greater. The more complex, outcome-response-stimulus update required for the slips-of-action test may facilitate more deliberative and careful responding, whereas the simpler baseline test may more strongly trigger automatic responses to stimuli. In line with this possibility and with recent data suggesting that automatic, habitual responses are prepared at short latencies and then replaced by new, goal-directed responses [47], all participants responded more quickly in the baseline than the slips-of-action test. We speculate that without slower, deliberative processing engaged when outcomes are explicitly devalued, women with BN have difficulty flexibly updating behavior and suppressing stimulus-response tendencies.

Finally, because it could be argued that the baseline test is more similar to a standard test of inhibitory control than the slips-of-action test is, we explored whether our baseline-test findings may be explained by response inhibition impairments in BN. Counter to this possibility, our BN group did not make significantly more commission errors on a separate go/no-go task, and performance on the go/no-go task was unrelated to baseline-test performance and parameters in women with BN. Overall, these results suggest that impaired performance on the baseline test may specifically reflect a deficit in repeatedly updating information about learned stimulus-response associations.

However, this deficit could potentially explain why individuals with BN show food-specific impairments in inhibitory control with medium to large effect sizes, but non-food-specific impairments in inhibitory control with small effect sizes [31]: Inhibitory control tasks used to study BN to date have all included static stimulus-response mappings, but those that instruct participants to withhold responses to images of food stimuli may present individuals with BN with the additional challenge of updating strong stimulus-response (i.e., food-go) associations (in Bayesian terms, strong priors) that were consolidated outside the task environment. Future research using batteries of food and non-food-specific versions of the instrumental learning and devaluation paradigm and inhibitory control tasks could begin to test whether impairments in the ability to adjust behavior to inhibit previously learned stimulus-response associations may help explain the more pronounced deficits detected on food-specific response inhibition tasks. Similarly, applications of computational modeling to cognitive flexibility tasks (e.g., [48]) may help clarify whether prior results suggesting perseverative behaviors and set-shifting deficits on such tasks in BN [44, 45] could be accounted for by deficits in the flexible updating of stimulus-response associations.

### Limitations

Several study limitations should be acknowledged. First, the samples were relatively small and included only adult females, and women with BN all engaged in self-induced vomiting as a compensatory behavior. The generalizability of our findings to males, younger age groups with shorter durations of illness, underrepresented ethnic groups, and non-purging individuals with BN is unknown. Replication of our findings in larger samples is needed. In addition, although our state measures suggest that hunger, fullness, metabolic state, and days since last menstrual period did not contribute to our group differences (Table 1; Supplement), future work may benefit from including standardized meals and assessment of estrogen and progesterone levels to more conclusively rule out potential influences of pre-task intake and hormonal factors on devaluation and belief updating. We did not assess for working memory capacity using a separate task, so we cannot completely rule out the possibility that group differences in working memory contributed to our results. However, accuracy scores from the knowledge check at the end of the task indicated that there were no group differences in explicit memory of stimulus-response, response-outcome, or stimulus-outcome associations, suggesting that this possibility is unlikely.

The baseline and slips-of-action tests used in the current study are also limited in their ability to model how individuals learn from changes in environmental contingencies because participants are explicitly, rather than implicitly, told about the changing value of outcomes or stimuli, and participants are not given trial-by-trial feedback on their performance. Both of these tests likely measure deficits in the engagement of control to adjust behavior following devaluation, but they cannot explicitly assess the extent to which altered performance is related to excessively strong development of habits versus impaired goal-directed control [29, 49]. Indeed, given that experimental tasks have not yet demonstrated habits as a function of behavioral repetition, there remains great debate as to how to measure habitual processes and whether they play an important role in performance on human behavioral tasks per se [50, 51]. Future work using a variety of measures and experimental paradigms (e.g., the Pavlovian-to-Instrumental Transfer task; the two-step decision-making task; the symmetrical outcome-revaluation task; self-report measures of behavioral automaticity) would be needed to better understand the interplay between goal-directed and habitual control that may underpin our findings [50]. Finally, our sensitivity analyses suggest our results were robust to a history of AN, current anxiety disorder, and psychotropic medication use, but because results were slightly changed when individuals with depression were excluded, larger studies explicitly comparing individuals with depression to individuals with BN (with and without depression) are needed.

### Clinical Implications

Despite these limitations, the current results may have important implications for understanding BN pathophysiology and treatment. Because our findings indicate that slower updates of stimulus-response associations may specifically underpin maladaptive bulimic symptoms, more prolonged exposure to stimuli that powerfully evoke binge eating and purging paired with response prevention may be helpful for individuals with more severe BN [3, 52]. Additionally, our participants with BN struggled with explicit stimulus devaluation, but data from experimental tasks in healthy individuals indicate that repeated practice of action stopping in response to valuable stimuli can implicitly devalue the stimuli [13]. This kind of practice via inhibitory control training programs has shown initial promise for binge eating [53, 54]. Examining the impact of such inhibition training programs on purging and framing these programs as a way to devalue symptom-prompting stimuli may prove fruitful. Finally, we found that women with BN did not have difficulty flexibly inhibiting behaviors that led to outcomes that they had explicitly learned were less valuable. As such, capitalizing on this intact ability by focusing on explicitly devaluing expected eating-disorder symptom outcomes, such as weight loss or thinness, and increasing the value of other outcomes, such as improved relationships, may be particularly effective for BN [6].

## Supplementary information

Supplemental Material

## Data Availability

The code used for computational modeling is available upon request.
