# Supplementary material for: Impaired belief updating and devaluation in adult women with bulimia nervosa
Source: Transl Psychiatry. 2023 Jan 6;13:2. doi: 10.1038/s41398-022-02257-6 (PMC9816187; doi:10.1038/s41398-022-02257-6)
Supplement: Supplementary file 1 — Supplemental Material [file 41398_2022_2257_MOESM1_ESM.docx]

Berner, Fiore et al.

**SUPPLEMENTARY MATERIAL**

**Supplementary Methods**

**Full Inclusion and Exclusion Criteria**

Participants were right-handed (1) females aged 18 and 35 and between 85 and 120% of the expected weight for their height based on the Metropolitan Life Insurance tables (2). Women with bulimia nervosa (BN) met *DSM-5* criteria (at least one objective bulimic episode and compensatory behavior per week for the past three months)(3), purged via self-induced vomiting (though other methods could additionally be endorsed), and were on a stable dose of all psychoactive medications for at least 4 weeks before study.

Healthy controls were excluded if they 1) ever met criteria for the diagnosis of any Axis I psychiatric disorder in their lifetime; 2) had any history of binge eating (including any history of an experience of “loss of control” over eating) or self-induced vomiting, laxative or diuretic misuse, or 3) used psychoactive or other medication known to affect mood or concentration in the last 3 months.

Women with BN were excluded if they had 1) any comorbid Axis I disorder apart from major depression, generalized anxiety disorder, social anxiety disorder, or panic disorder, 2) a primary diagnosis of any of these comorbidities; any psychopathology that might interfere with the ability to participate in the study (e.g., requiring inpatient hospitalization).

Universal exclusion criteria were: 1) current significant medical illness; 2) substance use disorder in the past 6 months; 4) pregnancy or planned pregnancy during the study period, or lactation; 5) current or past neurological disorder or organic brain syndrome or dementia; 6) history of a seizure or head trauma with loss of consciousness; 6) Full Scale IQ under 75.

Participants were screened for exclusionary psychiatric disorders using the Mini-International Neuropsychiatric Interview (M.I.N.I.; 4), and, for disorders not included in the M.I.N.I., the Structured Clinical Interview for *DSM-5* (SCID-5; 5). Included disorders (eating disorders, major depression, anxiety disorders, substance use disorder prior to 6 months ago) were more comprehensively assessed using the SCID-5. A modified structured interview used in neuroimaging studies of individuals with past eating disorders (6, 7) assessed eating disorder diagnostic history.

**Go/No-Go Task**

Participants were instructed to press the left or right button according to the direction of a stimulus arrow presented on the screen. A total of 11% of trials were no-go trials with upward pointing arrows. On these trials, participants were instructed to inhibit responding. The design of this particular go/no-go task controlled for the infrequency of no-go trials with “oddball” go trials (8-10). Oddball go trials (with arrows pointing either left or right at an upward, 45° angle) were also presented 11% of the time. All stimuli appeared on the screen for 600ms with a jittered inter-stimulus interval (ISI; 1.6-2.0s, mean ISI = 1.8s). A total of 416 trials were presented (with 48 no-go stimuli and 48 oddball go stimuli) across two blocks.

**Power Analysis**

Prior studies using this task have reported large effect sizes in other clinical populations (*d* = 1.32 (11); *d* = 0.91 (12)). A power analysis indicated that 30 participants per group would provide 99% power to detect large effects on the task (*d* = 0.90; *α* = .05, two-sided). We studied one additional HC participant, bringing the total number of subjects to 61 and yielding 80% power to detect moderate to large effects.

**Computational Modeling of Behavior**

**Reinforcement Learning Model**

We tested two potential computations of the prediction error $\left( O_{t}-V_{j,t} \right)$: one with a variable trial-by-trial outcome, and one with a fixed trial-by-trial outcome, as in (13). The variable outcome model used the actual values shown in the task after each choice selection. In this model, participants were rewarded with more points (between 0 and 5) the faster they correctly responded: $O=5$ (reaction times (RTs) <1 s, $O=4$ (RTs < 1.5 s), $O=3$ (RTs < 2s), $O=2$ (RTs < 2.5s), and $O=1$ for longer RTs. In the second version of the model, we assumed that participants were not sensitive to these variations in points, and as in (13) the value of the outcome $O$ was set to a constant of 5.

To transform values into choice selection probabilities [*p*(left_t_) and *p*(right_t_)], we used a softmax function adapted to account for subject-specific differences in perseverative and exploratory behavior, which could vary dynamically during the task. These behavioral phenotypes were captured by the use of two separate parameters affecting the temperature (i.e., the steepness) of the softmax transformation. These two parameters controlled the sensitivity to reinforcement and perseveration (*β* and *τ*, respectively)(13):

$p\left( i,t \right)=\frac{e^{\beta V_{t}^{i}+\tau C_{t}^{i}}}{\sum_{k=1}^{n} e^{\beta V_{t}^{k}+\tau C_{t}^{k}}}$ (1),

where *n* referred to the number of available responses (for the present task, *n*=2), and $C_{t}^{k}$ was assigned a value of 1 if the subject’s choice on the current trial (t) was a repetition of the choice selection on the immediately previous trial (*t*-1), and if not, $C_{t}^{k}$ was assigned a value of 0. Finally, *k* was used to indicate the vector (two dimensions) of the available responses (i.e., left or right).

Model fitting was performed using a Monte Carlo random search of parameters for the values of the learning rate (α), and for the two parameters controlling the softmax (*β* and *τ*). This method for the parameter regression was chosen because it allows for an unbiased exploration of the space of parameters, and it is not affected by discontinuities and local minima in the search for a global minimum error. We tested 200,000 randomly selected values for the three parameters, in the intervals [0, 1], [0, 10] and [-1, 1], respectively for α, β and τ. We computed the subject-specific trial-by-trial error associated with each combination of parameters as the logarithm of the predicted choice probability associated with the actual choice selections. The resulting error values were summed to assign an overall score to each set of parameters, allowing the selection of the best performing set.

**Bayesian Modeling**

With the Bayesian models, we aimed to estimate the subject-specific, trial-by trial subjective probabilities assigned to each action as the most likely to be associated with an outcome cue, given the input cue (i.e., Bayesian inference), or to be associated with an input cue, given the output cue (i.e., Bayesian inversion). For the first phase, the participants were instructed to maximize the number of rewarded output cue (*out_j_*) displays, per each input cue (*in*_j_) presentation. Therefore, the model estimated the subjective probability for each action (*a_left_ or a_right_*) to yield the desired output cue. The subsequent phases of the task (i.e., outcome-action learning, baseline, and slips-of-action) additionally required the participant to avoid responding to certain presented cues. Therefore, the model also assumed that the participants had to form independent beliefs concerning the possibility to pursue the task-related goals by either executing or not executing an action (*a_go_ or a_no-go_*), across all phases of the task. As a result, the trial-by-trial subjective probability that an action would yield a rewarded outcome, given an input cue, was equal to the joint probability assigned to the choice to perform an action or not and whether to press left or right. During the first phase, the prior beliefs for the current trial (*t*) were incrementally updated into posterior (*t+1*) beliefs according to Bayes rule, depending on subject-specific assumptions about the likelihood $\lambda=P\left( {out}_{j}|a_{i} \right)$ that the available action choices ($a_{i}$) would yield a desired outcome, as follows:

$P_{t+1,j}(a_{i})\propto P\left( {out}_{j}|a_{i} \right) P_{t,j}\left( a_{i} \right)$ (2).

Two independent computations were used to update left/right and go/no-go choice selections (see below for a step-by-step illustration of both update computations).

Importantly, this process of update relied on two key assumptions. First, because our participants’ behavior was characterized by subject-specific differences in the number of trials required to update beliefs, leading to errors in choice selections, we assumed subject-specific differences in the likelihoods, as if the environment were considered probabilistic instead of deterministic (cf. (14)). Therefore, the probabilities expressing the likelihoods for each action illustrated in equation 2 were assumed to vary in the continuous in the interval [0 1]. Second, we assumed that these likelihood distributions were fixed per participant. These likelihoods, in turn, determined the subject-specific trial-by-trial efficiency of the Bayesian update of priors into posteriors, and the modes of these distributions were set as free parameters in the model. A high precision for the likelihood is equivalent to a high value of the mode of the distribution (close to 1), whereas a low precision for the likelihood is equivalent to a low value of the mode of the distribution (close to 0.5).

For the first phase of the task, the set of parameters that best matched each participants’ choice selections was used to compare the fit of the RL and Bayesian learner models. For subsequent task phases, the Bayesian model relied on posteriors from the instrumental learning phase to guide the Bayesian belief update of *forward* associations in_j_-a_i_-out_j_, as described, as well as of *inverse* associations out_j_-a_i_-in_j_, for both left/right and go/no-go choice selections. The latter type was achieved by trading the roles of in_j_ and out_j_ in equation 2 and relying on two new parameters controlling the values of the mode in the likelihood distributions $P\left( {{in}_{j}|a}_{i} \right)$.

During the outcome-action learning test, we compared the consolidated beliefs about the a_i_-out_j_ associations achieved at the end of the first task phase (i.e., first-phase posteriors) with the actual participant performance to further refine the overall computation of the parameterization error.

In the baseline and slips-of-action tests, participants were shown updated information about which stimuli or outcomes were devalued after every 12 trials. For these tests, we assumed that the posterior beliefs about the go/no-go choice selection (consolidated by the end of the first two task phases, trial 144) were filtered in a one-shot decay, controlled by a hyper-parameter [d = 0.27; i.e. $P_{slips-of-action1}\left( a_{i} | {out}_{j} \right)=P_{144}\left( a_{i} | {out}_{j} \right)-0.27$; i.e., p(go) = 0.73, p(no-go) and ${P_{baseline1}\left( a_{i} | {in}_{j} \right)=P}_{144}\left( a_{i} | {in}_{j} \right)-0.27$)]), identical for all subjects. These filtered priors were then updated into posteriors, as previously described. After each new presentation of new devalued cues, the no-go probability would increase for devalued cues and decrease for valued cues. Left/right prior probabilities were not assumed to be updated, as the instructions provided during the slips-of-action and baseline tests only included information concerning the go/no-go choice.

**Step-by-Step Illustration of the Continuous Bayesian Belief Update.** At the start of the Bayesian process of belief updating (trial=1), the model establishes the initial priors at the start of the task as equal to:

$P_{1,j}(left)=P_{1,j}(right)=\frac{1}{2}$ (3)

$P_{1,j}(Go)=P_{1,j}(NoGo)=\frac{1}{2}$ (4)

In this example, we consider an agent who updates their beliefs assuming that a correct left/right choice will yield a desired outcome with 80% probability, whereas a correct go/no-go choice is associated with 95% probability to yield the desired outcome (i.e., the mode of the likelihood λ=[0.8, 0.2] and λ=[0.95, 0.05]). The probability assigned to the remaining, incorrect, action choices is complementary to the correct ones. Finally, we illustrate here only a sequence of action-outcomes associated with one single input cue, as follows: 1) go-left-no outcome; 2) go-right-outcome; 3) go-right-outcome. Note that, since the updates for the two action selection types are independent, there is the theoretical possibility of an update including no-go jointly with a left or right decision. However, this event never occurred in our study, as all participants expressed an action selection (i.e., in terms of the model, go-left or go-right).

For illustration purposes, the vectors of probability distribution represent the actions in the order [left, right] and [go, no-go].

Trial 1 prior: ${P\left( [left,right] \right)}_{t}=\left[ \frac{1}{2} \frac{1}{2} \right]$; ${P\left( [go,no-go] \right)}_{t}=\left[ \frac{1}{2} \frac{1}{2} \right]$

First update, go, left, no outcome:

${P\left( \left[ left, right \right] | {out}_{j} \right)}_{t+1}=\frac{\lambda* prior}{P({out}_{j})}=\frac{\left[ 0.2 0.8 \right].*[ \frac{1}{2} \frac{1}{2} ]}{\left( 0.2*\frac{1}{2}+0.8*\frac{1}{2} \right)}=\frac{[0.1 0.4]}{0.5}=[0.2, 0.8]$

${P\left( \left[ go, no-go \right] | {out}_{j} \right)}_{t+1}=\frac{\lambda* prior}{P({out}_{j})}=\frac{\left[ 0.2 0.8 \right].*[ \frac{1}{2} \frac{1}{2} ]}{\left( 0.2*\frac{1}{2}+0.8*\frac{1}{2} \right)}=\frac{[0.1 0.4]}{0.5}=[0.2, 0.8]$

Trial 2 prior: ${P\left( [left,right] \right)}_{t}=\left[ 0.2 0.8 \right]$; ${P\left( [go,no-go] \right)}_{t}=\left[ 0.2 0.8 \right]$

Second update, go, right, desired outcome:

${P\left( \left[ left, right \right] | {out}_{j} \right)}_{t+1}=\frac{\left[ 0.2 0.8 \right].*[ 0.2 0.8 ]}{\left( 0.2*0.2+0.8*0.8 \right)}=[0.0588, 0.9412]$

${P\left( \left[ go, no-go \right] | {out}_{j} \right)}_{t+1}=\frac{\left[ 0.8 0.2 \right].*[ 0.2 0.8 ]}{\left( 0.8*0.2+0.2*0.8 \right)}=[0.5, 0.5]$

Trial 3 prior: ${P\left( [left,right] \right)}_{t}=\left[ 0.0588, 0.9412 \right]$; ${P\left( [go,no-go] \right)}_{t}=\left[ 0.5 0.5 \right]$

Third update, go, right, desired outcome:

${P\left( \left[ left, right \right] | {out}_{j} \right)}_{t+1}=\frac{\left[ 0.2 0.8 \right].*[ 0.0588, 0.9412 ]}{\left( 0.2*0.0588+0.8*0.9412 \right)}=[0.0154, 0.9846]$

${P\left( \left[ go,no-go \right] | {out}_{j} \right)}_{t+1}=\frac{\left[ 0.8 0.2 \right].*[ 0.5 0.5 ]}{\left( 0.8*0.5+0.2*0.5 \right)}=[0.8, 0.2]$

**Assumption of Non-deterministic Likelihoods.** In Bayesian terms, the optimal likelihood $[\lambda=P\left( {out}_{j}|a_{i} \right)]$ in a deterministic, two-choice environment is equal to 0 or 1. Thus, the posterior probability after the Bayesian update [$P_{t+1}(a_{i})]$, equals 0 or 1 after only one piece of evidence is collected. Although the task used for this study is indeed deterministic in its *in_j_-a_i_-out_j_* associations, our participants’ behavior is characterized by subject-specific differences in the number of trials required to update beliefs, leading to errors in choice selections. We assumed that these behavioral differences indicated the presence of subject-specific learning rates, which were captured in the model by subject-specific differences in the assumed likelihoods, or the distributions of probabilities for the available events, as if the environment were considered probabilistic (cf.(14)). Therefore, the probabilities expressing the likelihoods for each action illustrated in equation 2 were assumed to vary in the continuous in the interval [0 1].

**Assumption of Fixed Left/Right and Go/No-Go Associations.** We assumed the probabilistic nature of the left/right and go/no-go associations were fixed per subject, indicating the subjects’ assumptions about the degree of the probabilistic nature of the task. These, in turn, determined the subject-specific trial-by-trial efficiency of Bayesian update of priors into posteriors. Distributions close to deterministic values resulted in fast updates (i.e., little evidence is required to establish an association), whereas distributions close to stochasticity (50% go, 50% no-go) resulted in very slow updates, as accumulated evidence was less informative under these conditions (cf. (14)).

**Bayesian Model Fitting.** The mode of the two likelihood distributions was used as free parameter in a random Monte Carlo search (10,000 randomly generated values were used for the two parameters (left/right, go/no-go), in the interval [0.5, 1]), assigning the complementary value to the remaining element in the distribution of probabilities in the likelihood. A high precision for the likelihood is equivalent to a high value of the mode of the distribution ($\lambda$; close to 1), whereas a low precision for the likelihood is equivalent to a low value of the mode of the distribution (close to 0.5). Therefore, the model simulated subject-specific belief updating, where the joint probabilities assigned to the choice selections (left/right, go/no-go) allowed us to estimate the subject-specific trial-by-trial error associated with each parametrization. The error was filtered trial-by-trial as log(error_t_), to assign a non-linear weight to the estimated probabilities for the actual choice selections.

During the outcome-action learning test, we compared the consolidated beliefs about the a_i_-out_j_ associations achieved at the end of the first task phase (i.e., first-phase posteriors) with the actual participant performance. Trial-by-trial log(error_t_) were computed as described for the first phase of the task and added to the overall computation of the parametrization error score.

The posteriors from the first two phases of the task were filtered by a hyper-parameter that controls the one-shot decay for go/no-go selections in the baseline and slips-of-action tests. This hyper-parameter was set as the mean of the values determined after an expanded parameter regression with a Monte Carlo search (10^5^ randomly generated sets of parameters). After the process of update, the subject-specific estimated probabilities assigned to the actual choice selection on a trial-by-trial basis were used to determine the error in the model-estimated choice probability for each randomly generated set of parameters, as follows:

$${error}_{par}= \sum max(-5,\log(\mathrm{choice}_{prob}))$$

Due to the presence of the logarithm, the parameters that provided the closest fit of the subjects’ behavior were estimated using an $argmax({error}_{par})$. The parameters were estimated independently for each phase of the task phases, as, for instance, the parameter regulating belief updating in the slips-of-action test had no effect on error estimations in the baseline test, and vice versa.

**Supplementary Results**

**Reinforcement Learning Model**

The best-fitting RL model included static reward values for correct responses (mean BIC for the model with more points for faster responses = 61.1 ± 17.5; mean BIC for the model with 5 points for correct responses = 58.2 ± 17.2 ; *t*(60) = 7.7, *p* < 0.001).

**Post-Hoc Exploratory Analyses**

Post-hoc exploratory analyses indicated that groups did not differ on any λ parameters from the Bayesian learner model on the first or second phases of the task (*p*s > 0.40).

A post-hoc follow-up analysis that collapsed performance across the baseline and slips-of-action tests indicated a group x valuation interaction (*B* = 9.74, *SE* = 3.73, *t* = 2.614, *p* = 0.010).

In *post-hoc* LMEs examining reaction time (RT) across tests, a main effect of task indicated that all participants responded more slowly to valued trials on the slips-of-action test than the baseline test (*B* = -63.19, *SE*= 14.59, *t* = -4.33, *p* < 0.001). Adding a group x task interaction did not improve the prediction of RT (*p* = 0.311). Similarly, across groups, when participants incorrectly responded to devalued trials, these responses were slower on the slips-of-action test than the baseline test (*B*= -99.33, *SE*= 36.08, *t* = -2.75, *p* = 0.008), and there was no group x task interaction (*p* = 0.298). Post-hoc Huber robust regressions indicated that baseline test and slips-of-action test devaluation sensitivity indices (DSI) were associated in the HC group (*z* = 0.123, *p* = 0.032), but were unassociated in the BN group (*p* = 0.078).

**Sensitivity Analyses**

As there was a statistically significant between-group difference in days since last menstrual period (main manuscript Table 1), we examined whether DSIs from the baseline and slips-of-action tests or the pace-of-update parameters from computational modeling of the baseline and slips-of-action tests were correlated with self-reported days since last menstrual period. The days since last menstrual period variable was unrelated to any of our task performance parameters of interest (in the full sample, in the BN group alone, and in the HC group alone; *p*s > 0.170).

Sensitivity analyses indicated that women with a history of AN did not differ from those without on baseline or slips-of-action test DSI or pace of update parameters (*p*s>0.357).

Analyses excluding women on medication (*n* = 5) indicated that there was no group x valuation effect for slips-of-action responses (*p* = 0.113), and no group differences in the slips-of-action test pace of update on the (*p* = 0.451), but there were still group x valuation effects for the baseline test (*B* = 8.65, *SE* = 2.88, *t* = 3.01, *p* = 0.003), and the BN group still showed a slower pace of update relative to controls on the baseline test (*W* = 530, *p* = 0.018).

Analyses excluding women with an anxiety disorder (*n* = 8) indicated that there were still group x valuation effects for the baseline test (*B* = 5.00, *SE* = 2.21, *t* = 2.26, *p* = 0.026), and the BN group still showed a slower pace of update on the baseline test (*W* = 452, *p* = 0.045). Group x valuation effects on the slips-of-action test remained non-significant (p = 0.077) and group differences in pace of update on the slips-of-action task remained non-statistically significantly different (*p* = 0.374) when those with anxiety were excluded.

Analyses excluding women with major depression (*n* = 8) indicated that there were still group x valuation effects for the baseline test (*B* = 4.68, *SE* = 2.18, *t* = 2.15, *p* = 0.034), but group differences in baseline-test pace of update were no longer statistically significant *(W* = 437, *p* = 0.084). Group differences in pace of update on the slips-of-action test remained non-statistically significantly different (*p* = 0.294) when those with depression were excluded, but group x valuation effects on the slips-of-action test became significant (*B* = 13.55, *SE* = 6.75, *t* = 2.01, *p* = 0.048).

**References**

1. Oldfield RC. The assessment and analysis of handedness: The Edinburgh Inventory. Neuropsychologia. 1971;9:97-113.

2. Metropolitan Life Insurance Company. New weight standards for men and women. Stat Bull Metrop Insur Co1959. p. 1-11.

3. American Psychiatric Association. Diagnostic and Statistical Manual of Mental Disorders: Fifth Edition (DSM-5). Washington. D.C.: American Psychiatric Association; 2013.

4. Sheehan DV, Lecrubier Y, Sheehan KH, Amorim P, Janavs J, Weiller E, et al. The Mini-International Neuropsychiatric Interview (M.I.N.I.): the development and validation of a structured diagnostic psychiatric interview for DSM-IV and ICD-10. The Journal of Clinical Psychiatry. 1998;59 (Suppl 20):22-33.

5. First M, Williams J, Karg R, Spitzer R. User’s Guide for the Structured Clinical Interview for DSM-5 Disorders, Research Version (SCID-5-RV). Arlington, VA, American Psychiatric Association. 2015.

6. Wierenga CE, Bischoff-Grethe A, Berner LA, Simmons AN, Bailer U, Paulus MP, et al. Increased anticipatory brain response to pleasant touch in women remitted from bulimia nervosa. Translational Psychiatry. 2020;10(1):236.

7. Berner LA, Simmons AN, Wierenga CE, Bischoff-Grethe A, Paulus MP, Bailer UF, et al. Altered anticipation and processing of aversive interoceptive experience among women remitted from bulimia nervosa. Neuropsychopharmacology. 2019;44(7):1265-73.

8. Rubia K, Smith AB, Woolley J, Nosarti C, Heyman I, Taylor E, et al. Progressive increase of frontostriatal brain activation from childhood to adulthood during event-related tasks of cognitive control. Human Brain Mapping. 2006;27(12):973-93.

9. Schmidt A, Walter M, Gerber H, Schmid O, Smieskova R, Bendfeldt K, et al. Inferior Frontal Cortex Modulation with an Acute Dose of Heroin During Cognitive Control. Neuropsychopharmacology. 2013;38(11):2231-9.

10. Smith AB, Taylor E, Brammer M, Toone B, Rubia K. Task-specific hypoactivation in prefrontal and temporoparietal brain regions during motor inhibition and task switching in medication-naive children and adolescents with attention deficit hyperactivity disorder. Am J Psychiatry. 2006;163(6):1044-51.

11. Gillan CM, Papmeyer M, Morein-Zamir S, Sahakian BJ, Fineberg NA, Robbins TW, et al. Disruption in the balance between goal-directed behavior and habit learning in obsessive-compulsive disorder. The American journal of psychiatry. 2011;168(7):718-26.

12. Delorme C, Salvador A, Valabrègue R, Roze E, Palminteri S, Vidailhet M, et al. Enhanced habit formation in Gilles de la Tourette syndrome. Brain. 2015;139(2):605-15.

13. Lim TV, Cardinal RN, Savulich G, Jones PS, Moustafa AA, Robbins T, et al. Impairments in reinforcement learning do not explain enhanced habit formation in cocaine use disorder. Psychopharmacology. 2019;236(8):2359-71.

14. Yu J-C, Fiore VG, Briggs RW, Braud J, Rubia K, Adinoff B, et al. An insula-driven network computes decision uncertainty and promotes abstinence in chronic cocaine users. European Journal of Neuroscience. 2020;52(12):4923-36.
